# Alteration of Resting Electroencephalography by Acute Caffeine Consumption in Early Phase Psychosis

**DOI:** 10.1177/15500594211057355

**Published:** 2021-11-22

**Authors:** Jenna N. Bissonnette, T-Jay Anderson, Katelyn J. McKearney, Philip G. Tibbo, Derek J. Fisher

**Affiliations:** 1 Department of Psychiatry, 3688 Dalhousie University, Halifax, Nova Scotia, Canada; 2 Department of Psychology, 3684 Mount Saint Vincent University, Halifax, Nova Scotia, Canada; 3 Department of Psychology & Neuroscience, 3688 Dalhousie University, Halifax, Nova Scotia, Canada; 4 Department of Psychology, 3690 Saint Mary's University, Halifax, Nova Scotia, Canada

**Keywords:** electroencephalography, schizophrenia, resting state, caffeine, alpha asymmetry

## Abstract

Individuals with schizophrenia use twice as much caffeine on average when compared to healthy controls. Knowing the high rates of consumption, and the potential negative effects of such, it is important we understand the cortical mechanisms that underlie caffeine use, and the consequences of caffeine use on neural circuits in this population. Using a randomized, placebo controlled, double-blind, repeated measures design, the current study examines caffeine's effects on resting electroencephalography (EEG) power in those who have been recently diagnosed with schizophrenia (SZ) compared to regular-using healthy controls (HC). Correlations between average caffeine consumption, withdrawal symptoms, drug related symptoms and clinical psychosis symptoms were measured and significant correlations with neurophysiological data were examined. Results showed caffeine had no effect on alpha asymmetry in the SZ group, although caffeine produced a more global effect on the reduction of alpha_2_ power in the SZ group. Further, those with more positive symptoms were found to have a greater reduction in alpha_2_ power following caffeine administration. Caffeine also reduced beta power during eyes closed and eyes open resting in HC, but only during eyes closed resting conditions in the SZ group. These findings provide a descriptive profile of the resting EEG state following caffeine administration in individuals with schizophrenia. The findings ultimately suggest caffeine does not affect alpha or beta power as readily in this population and a higher dose may be needed to achieve the desired effects, which may elucidate motivational factors for high caffeine use.

## Caffeine and Schizophrenia

Caffeine, an adenosine receptor antagonist, indirectly simulates the widespread release of dopamine, serotonin and noradrenaline throughout the cortex.^1^ One study reported that out of a sample of 146 community dwelling schizophrenia patients, 13% of them used 1000 mg of caffeine or more per day.^2^ Overall, individuals with schizophrenia used twice as much caffeine on average when compared to healthy controls.^2^ It has been suggested that the increase in dopamine activity caused by caffeine can worsen the positive symptoms of schizophrenia like hallucinations and delusions.^3^ Additionally, high rates of caffeine consumption can be problematic when coupled with specific antipsychotic and anxiolytic medications, like Clozapine.^4^ Although higher typical caffeine use in this population may result in better performance on complex executive functioning tasks,^5,6^ the effects of acute caffeine administration in schizophrenia have not been studied.^7^ Given the high rates of consumption and the potential negative side effects of caffeine in this population, it is important we deeply understand the cortical mechanisms that underlie caffeine use.

## EEG, the Resting State, and Caffeine

On average, individuals with schizophrenia have less resting alpha power, and augmented theta and delta power.^8,9,10,11,12^ These reported increases in slow-wave activity have previously been reported to be associated with negative symptoms.^8,11^ In addition to the overall reductions in alpha activity, individuals with schizophrenia typically exhibit a hemispheric asymmetry of more alpha power in the left hemisphere compared to the right.^13,14,15,16^ This alpha asymmetry was also found in a sample of individuals with early-phase psychosis.^13^ Healthy controls exhibit symmetrical alpha power and this symmetry in the alpha band is thought to be needed for effective information processing.^14^ The effects of caffeine on alpha asymmetry are not well documented, however other stimulants like nicotine have been shown to increase alpha asymmetry values.^17^

In moderate doses (approximately 200 mg), caffeine reduces fatigue and increases alertness and interest in relevant tasks.^18,19,20^ Previous studies have found benefits of caffeine across multiple cognitive processes like verbal working memory^21^, sustained attention^22^, and executive function.^23^ In a healthy population, following a moderate dose of caffeine (200-250 mg), beta^24,25^ and alpha power decrease in the frontal regions during eyes-open resting conditions.^24,25,26^ Conversely, during eyes closed resting, alpha_2_ power increases.^25,27^ This suggests caffeine may not increase arousal overall, but rather arousal of the central nervous system (CNS) may serve as a moderating factor for caffeine's effects.^28,29^ The effects of caffeine on the resting state in those with schizophrenia, however, are not well documented.

## The Current Study

To our knowledge, this will be the first study to examine caffeine's effects on resting state oscillatory band power and alpha asymmetry in a sample of those who have been diagnosed with schizophrenia within the past 5 years (within the early phase of psychosis). Using an early phase population will allow us to capture the effects of caffeine independent of medication or illness progression status. In accordance with previous findings of the effects of caffeine in a healthy population,^24,25,26,27^ we hypothesize that caffeine administration will increase alpha power during eyes-closed resting, and decrease alpha power during eyes-open resting in healthy controls. Further, we hypothesize that alpha asymmetry will decrease following the administration of caffeine in individuals with schizophrenia.

## Methods

### Participants

Participants consisted of 13 healthy controls (HC) (4 female, 9 male) between the ages of 19 to 35 (*M*  =  23.23, *SD*  =  4.28) as well as 14 individuals within the first five years of a primary diagnosis of schizophrenia (SZ) (3 female, 11 male) between the ages of 21 to 38 (*M*  =  27.43, *SD*  =  3.88). HC were recruited from the general public and SZ participants were recruited through the Nova Scotia Early Psychosis Program (NSEPP), where a diagnosis of schizophrenia was provided by the participants primary care physician. See table 1 for demographic characteristics of each participant group.

**Table 1.** Participant Characteristics.

	Early Phase Psychosis (SZ) (*n* = 14)	Healthy Controls (HC) (*n* = 13)
**Age (years)**	27.4 (3.9)	23.2 (4.3)
**Sex (M:F)**	11:3	9:4
**CCQ**	1490.0 (1201.4)	1250.1 (1398.8)
**CWSC**		
** **Caffeine session	4.4 (3.0)	2.5 (1.9)
** **Placebo session	5.1 (2.7)	3.1 (3.2)
**CDRS**		
** **Caffeine session	1.6 (1.0)	1.5 (0.9)
** **Placebo session	1.4 (0.6)	1.4 (0.7)
**Medication Status, %**	29% medicated	
**PANSS**		
Total	52.8 (13.2)	
Positive	12.9 (5.8)	
Negative	14.1 (5.1)	
General	25.9 (6.3)	
**PSYRATS**	13.3 (12.7)	
**BNSS**	21.6 (12.1)	

*Note.* The above table displays the average age, sex, caffeine consumption (CCQ), caffeine withdrawal (CWSC), and caffeine-related symptoms (CDRS) of each participant group as well as the clinical symptom scale scores of the SZ group.

#### Inclusion and Exclusion Criteria

All healthy controls had negative self-reported histories of psychiatric, medical and neurological illnesses. All SZ patients were judged to be clinically stable by their primary care physician and had no changes in their antipsychotic medication or symptoms in the past two months. Patients were also limited to use of an atypical antipsychotic (with the exclusion of clozapine due to its known interactions with caffeine^30^). SZ participants were excluded if any of the following criteria were met: co-morbid DSM-IV Axis I disorder; total PANSS score >65 reflecting an acute psychotic episode; or current history of drug abuse or dependence. Additionally, any participant was excluded if any of the following criteria were met: left handed; non-normal hearing and/or vision; history of a head injury resulting in a loss of consciousness; diagnosis of a neurological disorder; electro-convulsive therapy within the past year; significant cardiac illness, or extrapyramidal symptoms resulting in movement disorders which could affect ERP recordings.

Caffeine consumption (as measured by the Caffeine Consumption Questionnaire^31^) was recorded and complete non-users of caffeine were excluded due to the reported differences in behavioral and physiological effects between users and total non-users^32,33^. Beyond this requirement of at least some caffeine use, there were no minimum or maximum amounts of typical caffeine consumption for inclusion for this study.

### Design

The study used a randomized, placebo controlled, double-blind, repeated measures design. Each participant attended two sessions separated by minimum 24 h. In each session, either placebo or 200 mg of caffeine was administered. The order of drug administration was determined using counterbalancing so that half of the participants received caffeine during the first session and placebo during the second, while the remaining participants received the reverse order.

Caffeine pills contained 200 mg of caffeine and were physically identical to the pills used for placebo. This dose approximates the dose that would be consumed in an average 500 mL cup of drip coffee and was selected in accordance with previous studies that showed a moderate dose can exert widespread cerebral effects.^34^

### Procedure

Recording sessions were booked in the morning to ensure uniformity across sessions and to control for time-of-day effects^35^. Participants were required to abstain from illicit substances, alcohol, and cannabis from midnight the night before the session. They were also asked to abstain from any form of caffeine (coffee, tea, cola) from midnight until the testing session to ensure adequate clearing of caffeine given the maximum elimination half-life is 4.5 h.^36^ Verbal confirmation of abstinence was required.

Upon arriving at the lab, relevant questionnaires were given and drug treatment was administered at the same time as EEG set up. Directly following drug administration, withdrawal symptoms were measured using the caffeine withdrawal symptom checklist (CWSC). Thirty minutes after administration, EEG was recorded in a sound-attenuated chamber. Recordings included a 3-min eyes-open resting task (where the participant focused on a spot in front of them and relaxed with their eyes open) immediately followed by a 3-min eyes-closed resting task (where participants relaxed for three minutes with their eyes closed). At the end of the session, side effects of caffeine were assessed using the checklist of drug-related symptoms (CDRS). Informed consent was obtained from all participants and the study was cleared by the Nova Scotia Health Authority Research Ethics Board as well as the Mount Saint Vincent University Research Ethics Board.

### Questionnaires

Questionnaires used to measure caffeine usage and withdrawal variables are described in supplemental materials.

#### Psychotic Symptom Rating Scale (PSYRATS)

The PSYRATS can be further divided into the two subscales of auditory hallucinations and delusions^37^. The auditory hallucinations subscale of the PSYRATS was given to the SZ group to assess presence and severity of auditory hallucinations.

#### Brief Negative Symptom Scale (BNSS)

The BNSS was given to the SZ group to assess presence and severity of negative symptoms. The BNSS quantifies the following six specific domains of negative symptoms: distress, blunted affect, alogia, asociality, anhedonia and avolition.^38^

#### Positive and Negative Symptom Scale (PANSS)

The PANNS is a 30-item scale to assess the presence and severity of clinical psychotic symptoms.^39^ The PANSS includes 3 subscales of positive, general and negative symptoms, and scores can be derived for each subscale separately. a higher score indicates increased symptomology.

### Caffeine Consumption Questionnaire (CCQ)

The Caffeine Consumption Questionnaire^31^ was used to measure caffeine use. This scale gives a weekly total of 36 potential caffeine sources and the time of day which they are consumed making it a useful tool for providing an in-depth understanding of typical caffeine usage.

### Caffeine Withdrawal Symptom Checklist (CWSC)

Withdrawal symptoms were measured with the Caffeine Withdrawal Symptom Checklist which has been adapted from the Smoking Withdrawal Symptom Checklist.^40^ The CWSC consists of seven questions regarding the presence of different withdrawal symptoms (eg irritability, anxiety, depressed mood and desire to consume caffeine) that participants rate as either not present (0), mild (1), moderate (2) or severe (4).

### Checklist of Drug-Related Symptoms (CDRS)

A physical-symptom checklist used to measure nicotine-related symptoms^41^ was modified by removing itchiness near the nicotine patch. Participants rated the severity of their perceived caffeine-related symptoms (eg jitters, headache and nausea) on a 5-point scale: “no symptoms,” “mild symptoms,” “moderate symptoms,” “strong symptoms,” and “extreme symptoms”.

### EEG Recording Parameters

EEG recordings were digitally sampled at 500 Hz from an electrode cap with Ag + /Ag + -Cl- active electrodes at sixty-four scalp sites. Scalp sites were chosen according to the 10 to 10 system of electrode placement^42^ including three midline sites (frontal [Fz], central [Cz], parietal [Pz]); three left hemisphere (frontal [F3], central [C3], parietal [P3]) and three right hemisphere (frontal [F4], central [C4], parietal [P4]) electrode sites. Electrodes were also placed bilaterally on each mastoid, on the mid-forehead and nose (bipolar recordings of horizontal [HEOG] and vertical [VEOG] electro-oculogram activity were taken from supra-/sub orbital and external canthi sites, respectively). Electrode impendences were kept under 10 kΩ and all electrical signals were amplified with a bandpass of DC-250 Hz. Preprocessing included applying filters from 0.1 to 30 Hz with a notch filter at 60 Hz, segmentation into 2-s epochs (including 50% overlap) and artifact rejection of any epochs with electrical activity exceeding ± 75µV. For each condition, 2- second artifact-free epochs were subjected to a Fast Fourier Transform (FFT) algorithm with a Hanning window of 5%. EEG power averages were derived from each scalp site for each frequency band (delta: 1.5- 4.0 Hz; theta: 4.0-8.0 Hz; alpha_1_: 8.0- 10.5 Hz; alpha_2_: 10.5-13.0 Hz; beta: 13.0- 20.0 Hz) and then natural log-transformed (ln) offline. The scalp sites that were examined for this study were left (F3) and right (F4) frontal regions as well as left (P3) and right (P4) parietal regions. These sites have successfully been used previously while examining the effects of psychostimulants on the resting state^43,44,45^ An additional computation of alpha asymmetry was taken by subtracting the left hemisphere alpha power scores from the right hemisphere alpha power scores for both frontal and parietal regions.^46^

### Statistical Analysis

All statistical analyses were done using the Statistical Packages for Social Sciences (SPSS; IBM Corp. Armonk NY). Natural log-transformed EEG power measures for each band were separately analyzed using a repeated-measures general linear model (GLM) where treatment (caffeine vs. placebo), region (frontal vs. parietal) and site (left vs. right) were within-subject factors and group (SZ vs. HC) was used as a between-subject factor. In the case that the GLM indicated significant effects (*p* < .05), planned pairwise comparisons were done to determine main effects using a correction for multiple comparisons. For purposes of control analysis, spearman's rho bivariate correlations were completed between PSYRATS, PANSS, and BNSS scores. Additionally, spearman's rho bivariate correlations between total scores on the PSYRATS, PANSS, BNSS total scores, and difference in power between caffeine and placebo sessions at F3, F4, P3 and P4 for each oscillatory band were done for the SZ group. In both groups, spearman's rho bivariate correlations between scores on the CCQ, CWSC and CDRS and oscillatory power at F3, F4, P3 and P4 were completed for both caffeine and placebo sessions separately.

Alpha asymmetry values for each region (frontal, parietal) were separately analyzed using a repeated measures analysis of variance (ANOVA) where drug (caffeine vs. placebo) served as a within-subjects factor and group (HC vs. SZ) served as a between-subjects factor.

## Results

There were no main effects of group for any oscillatory band power. For main effects of site and region, as well as correlations with caffeine usage and withdrawal variables, and findings of individual electrode sites where interaction effects are significant, refer to supplemental materials.

### Eyes-Open Resting EEG

#### Delta & Theta

There were no significant main effects or interaction effects on delta or theta power.

#### Alpha_1_

There was a main effect of drug where, compared to placebo (*M*  =  1.25, *SD*  =  0.50), caffeine (*M*  =  1.13, *SD*  =  0.48) resulted in lower alpha_1_ power (*p*  =  .003, *Hedges’ g*  =  0.24). When the effect of group was considered, it was found that this reduction in alpha_1_ power was present in both the HC (*M_caf_*  =  1.07, *SD_caf_*  =  0.49, *M_pla_*  =  1.19, *SD_pla_*  =  0.54, *p*  =  .021, *Hedges’ g*  =  0.23) and SZ (*M_caf_*  =  1.19, *SD_caf_*  =  0.47, *M_pla_*  =  1.30, *SD*_pla_  =  0.45, *p*  =  .037, *Hedges’ g*  =  0.24) groups. Followed up further to include region, it was found that in the HC group, caffeine (*M*  =  1.06, *SD*  =  0.43) produced significantly lower alpha_1_ power than placebo (*M*  =  1.20, *SD*  =  0.50) at the frontal region (*p*  =  .007, *Hedges’ g*  =  0.30), but not the parietal region (*p*  =  .096). Similarly, in the SZ group, caffeine (*M*  =  1.20, *SD*  =  0.42) resulted in significantly lower alpha_1_ power than placebo (*M*  =  1.31, *SD*  =  0.43) in the frontal region (*p*  =  .029, *Hedges’ g*  =  0.26), but not the parietal region (*p*  =  .089).

#### Alpha_2_

**Figure 1.**
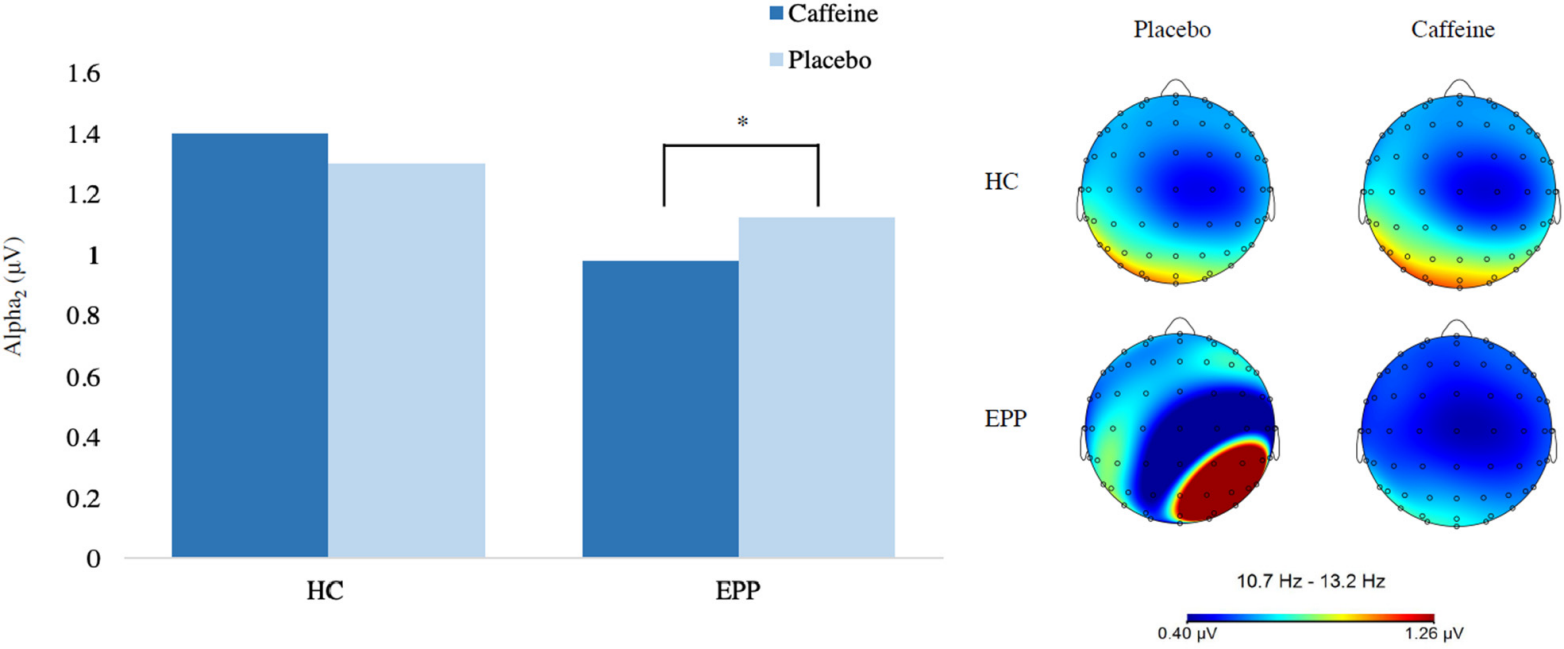
Reduction of parietal Alpha2 in early phase psychosis following caffeine administration. *Note.* The above figure demonstrates the reduction of parietal alpha_2_ power in the early phase psychosis (EPP) group, but not the healthy control (HC) group during eyes-open resting conditions.

There was a main effect of drug where, compared to placebo administration (*M*  =  0.91, *SD*  =  0.36), caffeine (*M*  =  0.81, *SD*  =  0.39) resulted in reduced alpha_2_ power (*p*  =  .002, *Hedges’ g*  =  0.27). There was also a drug-by-group interaction (Figure 1) where caffeine (*M*  =  0.81, *SD*  =  0.33) resulted in lower alpha_2_ power than placebo (*M*  =  0.94, *SD*  =  0.29) in the SZ group only (*p*  =  .005, *Hedges’ g*  =  0.42). Followed up further to examine region, caffeine (*M*  =  0.73, *SD*  =  0.33) resulted in lower alpha_2_ power than placebo (*M*  =  0.86, *SD*  =  0.33) at the frontal region in the HC group (*p*  =  .004, *Hedges’ g*  =  0.39), while in the SZ group, caffeine resulted in lower alpha_2_ power in both the frontal (*M_caf_*  =  0.79, *SD_caf_*  =  0.27, *M_pla_*  =  0.93, *SD_pla_*  =  0.25, *p*  =  .002, *Hedges’ g*  =  0.54) and parietal (*M_caf_*  =  0.84, *SD_caf_*  =  0.38, *M_pla_*  =  0.94, *SD_pla_*  =  0.32, *p*  =  .027, *Hedges’ g*  =  0.28) regions.

#### Beta

There was a main effect of drug where, compared to placebo (*M*  =  1.62, *SD*  =  0.27), caffeine (*M*  =  1.52, *SD*  =  0.28) resulted in lower beta power (*p*  =  .005, *Hedges’ g*  =  0.36). There was a group-by-drug interaction where compared to placebo (*M*  =  1.62, *SD*  =  0.26), caffeine (*M*  =  1.49, *SD*  =  0.31) resulted in lower beta power in the HC group only (*p*  =  .008, *Hedges’ g*  =  0.45). When followed up to include region, caffeine significantly reduced beta power compared to placebo at both the frontal (*M_caf_*  =  1.34, *SD_caf_*  =  0.28, *M_pla_*  =  1.70, *SD_pla_*  =  0.23, *p*  =  .003, *Hedges’ g*  =  1.41; see Figure 1) and parietal (*M_caf_*  =  1.45, *SD_caf_*  =  0.38, *M_pla_*  =  1.54, *SD_pla_*  =  0.27, *p*  =  .041, *Hedges’ g*  =  0.27) regions in the HC group.

### Eyes-Closed Resting EEG

#### Delta, Theta, & Alpha_1_

There were no significant main effects or interaction effects on alpha_1_, delta or theta power.

#### Alpha_2_

**Figure 2.**
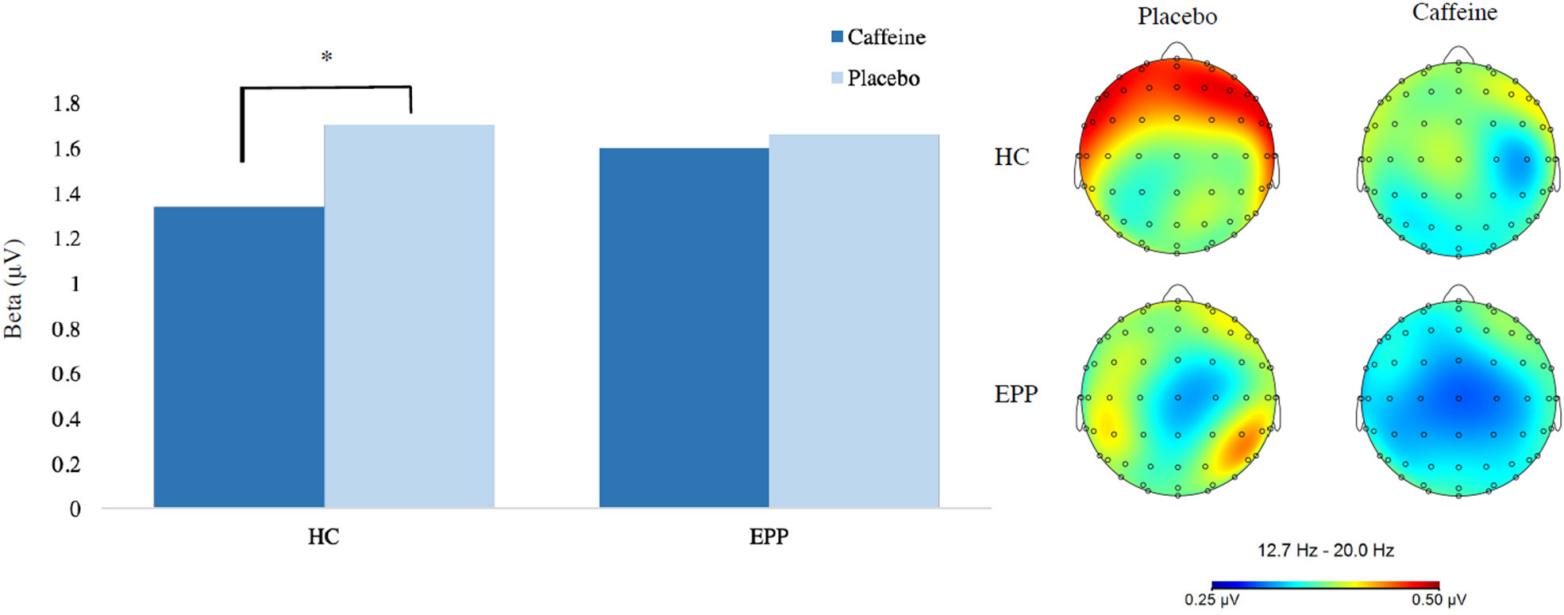
Reduction of frontal Beta in healthy controls following caffeine administration. *Note.* The above figure demonstrates the reduction of frontal beta power following caffeine administration in the healthy control (HC) group, but not the early phase psychosis (EPP) group during eyes-closed resting conditions.

Although there was no significant main effect of drug, there was a trend with a small effect size for a drug-by-group interaction (*p*  =  .064, *Hedges’ g*  =  0.27), where in the SZ group only, caffeine (*M*  =  1.08, *SD*  =  0.32) resulted in lower alpha_2_ power than placebo (*M*  =  1.17, *SD*  =  0.34). There was also a significant group-by-drug-by-region interaction where in the SZ group, caffeine (*M*  =  0.98, *SD*  =  0.29) resulted in lower alpha_2_ power than placebo (*M*  =  1.12, *SD*  =  0.34) in the frontal region (*p*  =  .008, *Hedges’ g*  =  0.44; see Figure 2), this reduction was not significant at the parietal region (*p*  =  .320).

#### Beta

There was a main effect of drug (*p*  =  .030, *Hedges’ g*  =  0.26) where caffeine (*M*  =  1.63, *SD*  =  0.32) resulted in less beta power than placebo (*M*  =  1.71, *SD*  =  0.30) overall. There was also a significant group-by-drug-by-region interaction (Figure 2), where in the HC group, caffeine (*M*  =  1.61, *SD*  =  0.32) resulted in lower beta power than placebo (*M*  =  1.71, *SD*  =  0.34) at the frontal region (*p*  =  .030, *Hedges’ g*  =  0.30), but not the parietal region (*p*  =  .357). Similarly, in the SZ group, caffeine (*M*  =  1.64, *SD*  =  0.25) resulted in less beta power than placebo (*M*  =  1.74, *SD*  =  0.25) at the frontal region (*p*  =  .036, *Hedges’ g*  =  0.40), but not the parietal region (*p*  =  .335).

### Alpha Asymmetry

There were no significant differences found for alpha asymmetry values between sessions in either group at either region.

### Correlations

#### PSYRATS

PSYRATS scores were not significantly correlated with BNSS or PANSS scores. Auditory hallucination subscale scores were significantly correlated with the difference in beta power between sessions in the frontal region (F3) during eyes closed, (*r*  =  -.56, *p*  =  .036) and eyes open resting (*r*  =  -.82, *p*  =  .000). The difference in alpha_2_ power between sessions was also associated with PSYRATS scores at F3 (*r*  =  -.87, *p*  =  .007), F4 (*r*  =  -.66, *p*  =  .010), and P4 (*r*  =  -.56, *p*  =  .038). Indicating that the increase in auditory hallucinations is associated with a lesser effect of caffeine on resting beta and alpha_2_ power.

#### BNSS

BNSS scores were not significantly correlated with PSYRATS or PANSS scores. There were significant positive correlations between the difference in theta power between sessions and BNSS scores at F3 (*r*  =  .78, *p*  =  .001), F4 (*r*  =  .65, *p*  =  .011) indicating that those with increased negative psychotic symptoms display an increased effect of caffeine on resting theta power.

#### PANSS

PANSS total scores were significantly correlated with PANSS positive (*r*  =  .56, *p*  =  .012) and negative (*r*  =  .79, *p*  =  .000) sub-scale scores, but not BNSS or PSYRATS scores. There was a significant correlation between parietal theta power (P4) and PANSS total scores (*r*  =  .56, *p*  =  .036). When examining only the positive symptom subscale, this correlation remained significant (*r*  =  .54, *p*  =  .045). This indicates that increased positive symptoms are related to an increased effect of caffeine on resting theta power. There were no significant relationships between the negative symptoms subscale of the PANSS and any oscillatory band power.

#### Caffeine Consumption Questionnaire (CCQ)

During eyes-open resting conditions, the HC group had significant correlations between their total CCQ scores and delta power at electrode sites F3 (*r*  =  -.63, *p*  =  .021), F4 (*r*  =  -.66, *p*  =  .014), P3 (*r*  =  -.74, *p*  =  .044), and P4 (*r*  =  -.63, *p*  =  .021), where increased typical caffeine use was associated with reduced delta power at rest in both drug conditions. This relationship also existed in eyes closed resting conditions at electrode sites F3 (*r*  =  -.56, *p*  =  .049), P3 (*r*  =  -.62, *p*  =  .021), and P4 (*r*  =  -.69, *p*  =  .010). However, this negative relationship between higher typical caffeine intake and reduced delta power was not present in the SZ group.

#### Caffeine Withdrawal Symptoms Checklist (CWSC)

There were so significant correlations between caffeine withdrawal symptoms indexed by the CWSC and oscillatory band power in the placebo or caffeine session for the HC group. In the SZ group, there were significant positive correlations between delta power at P4 (*r*  =  .56, *p*  =  .036), and theta power at F4 (*r*  =  .62, *p*  =  .018), P3 (*r*  =  -.56, *p*  =  .049), and P4 (*r*  =  -.56, *p*  =  .049) and CWSC scores, indicating that in the SZ group there were increased slow wave theta and delta power when caffeine withdrawal symptoms were higher.

#### Checklist of Drug-Related Symptoms (CDRS)

There were no significant correlations between caffeine related symptoms following drug administration and oscillatory band power in either group.

## Discussion

The current study did not find any main effects for group in regard to any of the oscillatory bands. This is against previous reports^47,48^ that alpha power is lower in individuals with schizophrenia compared to healthy controls. One possible explanation for these inconsistencies is that the previous studies have been completed with chronically ill and non-medicated samples, where the current sample was within the early phase of the illness and consisted of mixed but primarily medicated individuals. This could also explain why the finding of significant correlations between alpha power and negative symptoms were not replicated. A later study reported that reduced alpha power in schizophrenia may not be a reliable finding due to a wide range of data collection and analysis methods across studies.^49^

During the eyes-closed resting task, caffeine administration had no effect on alpha_2_ power in the HC group. However, in the SZ group, caffeine reduced alpha_2_ power in the frontal region. Additionally, during eyes-open resting, alpha_2_ power was reduced in the frontal and parietal region in the SZ group, but only in the frontal region of the HC group. This signifies an overall increased effect on alpha_2_ power in individuals with schizophrenia. Further, the opposite effect was seen with beta power (a decreased effect of caffeine on resting beta power in those with schizophrenia). Ultimately, these results suggest a moderate dose of caffeine differentially effects alpha and beta power in individuals with schizophrenia compared to healthy controls.

Increased auditory hallucinations were associated with a smaller effect on alpha_2_ power in SZ. It has been previously reported that those with increased positive symptoms have reduced resting alpha power without drug intervention compared to those with lower positive symptoms.^50^ It is possible that because those with greater positive symptoms have less alpha power to begin with, caffeine's reducing effects on alpha are not as strong in those with prevalent auditory hallucinations. The current results suggest perhaps a moderate dose of caffeine does not affect alpha and beta power as readily in those with auditory hallucinations and, therefore, a greater dosage is needed to experience the same effects. This could offer a possible theory underlying the higher rates of caffeine consumption in these subpopulations, where higher doses are required to achieve effects.

Change in theta power between sessions was significantly correlated with negative symptoms where caffeine had a larger effect on theta power in those with greater negative symptoms. Theta power is higher in the those with greater negative symptoms,^8^ therefore it is possible that the larger decrease we found was because there is more theta power to reduce to a baseline in those individuals. It was also found that higher positive symptoms (indexed by the PANSS positive symptom subscale) were also related to more change in theta power between sessions. Frontal theta power has been shown to be positively correlated with subjective sleepiness ratings.^51^ Therefore, these relationships where those with greater psychosis symptomology, both positive and negative, display a greater reduction in theta power could represent a stronger experience of the energizing effects of caffeine, and could provide an explanation of motivations for high caffeine use in this population.

### Limitations and Future Directions

First, the current patient sample contained both medicated and non-medicated individuals. The exact dose of antipsychotic medication being taken by the medicated patients is unknown. Future studies should consider examining the possible effect of antipsychotic medications on this data. Second, the lack of clinical assessment to screen out psychiatric diagnoses of participants in the healthy control group is a primary limitation of the current study. Similarly, the lack of clinical assessment to confirm schizophrenia diagnosis in our patient group is also a limitation of this study design.

Regarding the evaluation of psychosis symptoms in our patient group, the PANSS scores were assessed using the 3 sub-scale scores of positive, negative and general symptoms. However, it may have been more appropriate to use the 5-factor model that yields depressed, excited and disorganized factors.^38,52^ Future studies should consider correlations with these factor scores in addition to the positive and negative symptoms scores derived from the PANSS. Similarly, using the two-factor model of the BNSS (expressive and experiential factors) could give better insight into which specific negative symptoms are related to spectral power.

### Conclusion

Ultimately, it seems as though caffeine has different, and in some cases enhanced, effects on the resting EEG state in those with schizophrenia compared to healthy controls. This could elucidate motivational factors for the high caffeine use we see in this population.

## Supplemental Material

sj-docx-1-eeg-10.1177_15500594211057355 - Supplemental material for Alteration of Resting Electroencephalography by Acute Caffeine Consumption in Early Phase Psychosis Click here for additional data file. Supplemental material, sj-docx-1-eeg-10.1177_15500594211057355 for Alteration of Resting Electroencephalography by Acute Caffeine Consumption in Early Phase Psychosis by Jenna N. Bissonnette, T-Jay Anderson, Katelyn J. McKearney, Philip G. Tibbo and Derek J. Fisher in Clinical EEG and Neuroscience
