# Supplementary material for: Alteration of Resting Electroencephalography by Acute Caffeine Consumption in Early Phase Psychosis
Source: Clin EEG Neurosci. 2021 Nov 22;53(4):326–34. doi: 10.1177/15500594211057355 (PMC9174578; doi:10.1177/15500594211057355)
Supplement: sj-docx-1-eeg-10.1177_15500594211057355 - Supplemental material for Alteration of Resting Electroencephalography by Acute Caffeine Consumption in Early Phase Psychosis [file sj-docx-1-eeg-10.1177_15500594211057355.docx]

**Supplemental Material**

**Main Effects of Region and Site**

Main effects for site and region are reported in table 1 and table 2.

**Table 1**

*Main Effects of Region on Oscillatory Band Power*

|  |  | Frontal | | Parietal | |  |
| --- | --- | --- | --- | --- | --- | --- |
| EO |  | *M*(µV^2^) | *SD* | *M*(µV^2^) | *SD* | *p* |
|  | Alpha_1_ | 1.19 | 0.45 | 1.19 | 0.53 | .822 |
|  | Alpha_2_ | 0.83 | 0.30 | 0.90 | 0.45 | .135 |
|  | Beta | 1.62 | 0.25 | 1.52 | 0.29 | .000** |
|  | Delta | 1.44 | 0.23 | 1.21 | 0.29 | .000** |
|  | Theta | 1.74 | 0.36 | 1.47 | 0.39 | .000** |
| EC |  |  |  |  |  |  |
|  | Alpha_1_ | 1.61 | 0.48 | 1.68 | 0.58 | .111 |
|  | Alpha_2_ | 1.10 | 0.44 | 1.27 | 0.50 | .001** |
|  | Beta | 1.67 | 0.29 | 1.67 | 0.33 | .851 |
|  | Delta | 1.53 | 0.23 | 1.34 | 0.29 | .000** |
|  | Theta | 1.89 | 0.36 | 1.73 | 0.43 | .000** |

Note: *M* and *SD* are used to represent mean and standard deviation, respectively.

* indicates *p* < .05.

** indicates *p* < .01.

**Table 2**

*Main Effects of Site on Oscillatory Band Power*

|  |  | Left | | Right | |  |
| --- | --- | --- | --- | --- | --- | --- |
| EO |  | *M*(µV^2^) | *SD* | *M*(µV^2^) | *SD* | *p* |
|  | Alpha_1_ | 1.17 | 0.49 | 1.21 | 0.49 | .044* |
|  | Alpha_2_ | 0.85 | 0.38 | 0.87 | 0.39 | .136 |
|  | Beta | 1.55 | 0.28 | 1.59 | 0.27 | .025* |
|  | Delta | 1.31 | 0.30 | 1.34 | 0.30 | .045* |
|  | Theta | 1.59 | 0.43 | 1.62 | 0.43 | .027* |
| EC |  |  |  |  |  |  |
|  | Alpha_1_ | 1.63 | 0.534 | 1.66 | 0.53 | .015* |
|  | Alpha_2_ | 1.16 | 0.44 | 1.21 | 0.46 | .005** |
|  | Beta | 1.65 | 0.35 | 1.69 | 0.35 | .012* |
|  | Delta | 1.42 | 0.32 | 1.45 | 0.31 | .010* |
|  | Theta | 1.80 | 0.44 | 1.82 | 0.44 | .084 |

Note: *M* and *SD* are used to represent mean and standard deviation, respectively.

* indicates *p* < .05.

** indicates *p* < .01.

1. **Examination of Individual Electrode Sites Where Interaction Effects Were Found**

**Eyes Open Resting**

***Alpha1***

It was determined that in the HC group, caffeine resulted in reduced alpha_1_ power compared to placebo at electrode sites F3, F4, and in the SZ group caffeine resulted in reduced alpha_1_ power at electrode sides F3, F4, and P3 (see table 3).

**Table 3**

*Reduction of Alpha_1_ Power at Electrode Sites Following Caffeine Administration.*

|  |  | Caffeine | | Placebo | |  |
| --- | --- | --- | --- | --- | --- | --- |
| Group | Site | *M* (µV^2^) | *SD* | *M* (µV^2^) | *SD* | *p* |
| HC | F3 | 1.06 | 0.45 | 1.20 | 0.53 | .013 |
|  | F4 | 1.05 | 0.44 | 1.20 | 0.49 | .005 |
| SZ | F3 | 1.19 | 0.42 | 1.30 | 0.42 | .032 |
|  | F4 | 1.22 | 0.43 | 1.32 | 0.44 | .036 |
|  | P3 | 1.12 | 0.55 | 1.27 | 0.30 | .029 |

Note: *M* and *SD* are used to represent mean and standard deviation, respectively.

***Alpha2***

It was determined that caffeine resulted in reduced alpha_2_ power at F3 and F4 electrode sites in the HC group, and electrode sites F3, F4 and P4 in the SZ group (see table 4).

**Table 4**

*Reduction of Alpha*_2_ *Power at Electrode Sites Following Caffeine Administration.*

|  |  | Caffeine | | Placebo | |  |
| --- | --- | --- | --- | --- | --- | --- |
| Group | Site | *M* (µV^2^) | *SD* | *M* (µV^2^) | *SD* | *p* |
| HC | F3 | 0.72 | 0.34 | 0.87 | 0.36 | .003 |
|  | F4 | 0.74 | 0.34 | 0.87 | 0.32 | .008 |
| SZ | F3 | 0.78 | 0.27 | 0.92 | 0.26 | .003 |
|  | F4 | 0.80 | 0.28 | 0.93 | 0.26 | .003 |
|  | P4 | 0.86 | 0.40 | 0.97 | 0.34 | .023 |

Note: *M* and *SD* are used to represent mean and standard deviation, respectively.

***Beta***

Examination of individual electrode sites determined caffeine resulted in lower beta power than placebo at electrode sites F3 (*M_caf_* = 1.53, *SD_caf_* = 0.29, *M_pla_* = 1.68, *SD_pla_* = 0.25, *p* = .004, *Hedges’ g* = 0.55) and F4 (*M_caf_* = 1.55, *SD_caf_* = 0.28, *M_pla_* = 1.71, *SD_pla_* = 0.22, *p* = .004, *Hedges’ g* = 0.64), while the parietal electrode sites, P3 and P4, demonstrated trends towards lower beta power but did not reach significance.

**Eyes Closed Resting**

***Alpha2***

Reduction in alpha_2_ power following caffeine administration was present at both F3 (*M_caf_* = 0.99, *SD_caf_* = 0.31, *M_pla_* = 1.12, *SD_pla_* = 0.37, *p* = .047, *Hedges’ g* = 0.38) and F4 (*M_caf_* = 0.98, *SD_caf_* = 0.27, *M_pla_* = 1.12, *SD_pla_* = 0.31, *p* = .048, *Hedges’ g* = 0.48).

***Beta***

Ultimately, caffeine resulted in reduced beta power at electrode sites F3 and F4 in both the HC and SZ groups (Table 5).

**Table 5**

*Reduction of Beta Power at Significant Electrode Sites Following Caffeine Administration.*

|  |  | Caffeine | | Placebo | |  |
| --- | --- | --- | --- | --- | --- | --- |
| Group | Site | *M* (µV^2^) | *SD* | *M* (µV^2^) | *SD* | *p* |
| HC | F3 | 1.39 | 0.32 | 1.70 | 0.35 | .023 |
|  | F4 | 1.62 | 0.33 | 1.72 | 0.34 | .043 |
| SZ | F3 | 1.63 | 0.24 | 1.72 | 0.25 | .030 |
|  | F4 | 1.65 | 0.27 | 1.75 | 0.26 | .049 |

Note: *M* and *SD* are used to represent mean and standard deviation, respectively.
